# An observational study on factors associated with ICU mortality in Covid-19 patients and critical review of the literature

**DOI:** 10.1038/s41598-023-34613-x

**Published:** 2023-05-13

**Authors:** Athina Lavrentieva, Evangelos Kaimakamis, Vassileios Voutsas, Militsa Bitzani

**Affiliations:** 1st Intensive Care Unit, “G. Papanikolaou” General Hospital, 57010 Thessaloniki, Greece

**Keywords:** Infectious-disease diagnostics, Virology, Risk factors, Outcomes research, Prognosis, Infectious diseases, Viral infection, Respiratory distress syndrome

## Abstract

The novel pandemic caused by SARS-CoV-2 has been associated with increased burden on healthcare system. Recognizing the variables that independently predict death in COVID-19 is of great importance. The study was carried out prospectively in a single ICU in northern Greece. It was based on the collection of data during clinical practice in 375 adult patients who were tested positive for SARS-CoV-2 between April 2020 and February 2022. All patients were intubated due to acute respiratory insufficiency and received Invasive Mechanical Ventilation. The primary outcome was ICU mortality. Secondary outcomes were 28-day mortality and independent predictors of mortality at 28 days and during ICU hospitalization. For continuous variables with normal distribution, t-test was used for means comparison between two groups and one-way ANOVA for multiple comparisons. When the distribution was not normal, comparisons were performed using the Mann–Whitney test. Comparisons between discrete variables were made using the x^2^ test, whereas the binary logistic regression was employed for the definition of factors affecting survival inside the ICU and after 28 days. Of the total number of patients intubated due to COVID-19 during the study period, 239 (63.7%) were male. Overall, the ICU survival was 49.6%, whereas the 28-day survival reached 46.9%. The survival rates inside the ICU for the four main viral variants were 54.9%, 50.3%, 39.7% and 50% for the Alpha, Beta, Delta and Omicron variants, respectively. Logistic regressions for outcome revealed that the following parameters were independently associated with ICU survival: wave, SOFA @day1, Remdesivir use, AKI, Sepsis, Enteral Insufficiency, Duration of ICU stay and WBC. Similarly, the parameters affecting the 28-days survival were: duration of stay in ICU, SOFA @day1, WBC, Wave, AKI and Enteral Insufficiency. In this observational cohort study of critically ill COVID-19 patients we report an association between mortality and the wave sequence, SOFA score on admission, the use of Remdesivir, presence of AKI, presence of gastrointestinal failure, sepsis and WBC levels. Strengths of this study are the large number of critically ill COVID-19 patients included, and the comparison of the adjusted mortality rates between pandemic waves within a two year-study period.

## Introduction

The novel pandemic caused by SARS coronavirus-2 (SARS-CoV-2) has been associated with increased burden on healthcare system capacities worldwide^1^. Patients with COVID-19 have high hospital and intensive care unit (ICU) admission rates^2^. Mechanical ventilation continues to be the cornerstone of management of COVID-19 patients with life-threatening acute respiratory distress syndrome (ARDS); approximately one-third of these patients require some form of mechanical ventilation^3–5^. Outcome data for patients with COVID-19 receiving invasive mechanical ventilation have varied considerably. Meta-analyses of patients requiring ICU admission and mechanical ventilation referred to mortality rates of 40–45%^6^. Understanding the factors associated with mortality in patients requiring critical care and mechanical ventilation is highly relevant. Early studies on COVID-19 highlighted the presence of several factors such as patients’ baseline characteristics (hypertension, diabetes mellitus, obesity) and increased inflammatory markers levels as predictors of poor outcomes^2,7–14^.

Recently published data demonstrated that applying novel risk predictive models via specific machine learning algorithms could provide clinicians with more precise diagnostic tools, identifying patients at higher risk of complications such as health care-associated infections and may play a crucial role in improving patients’ outcome^15,16^.

However, the mortality of critically ill patients with COVID-19 receiving invasive mechanical ventilation has been reported to be highly variable. Causes of this inconsistency likely include the heterogeneity in the management of these patients and in the presentation of outcome data^3,17,18^.

It has been assumed that management of acute hypoxaemic respiratory failure and ARDS in COVID-19 patients largely mirrors that for non-COVID-19 etiologies. Recent data have shown that ARDS related to COVID-19 shares common pathophysiological and clinical features with ARDS of other causes; treatment goals of ARDS seem to be similar with goals for non-Covid-19 etiologies^19,20^. However, patients undergoing invasive mechanical ventilation have significantly longer mechanical ventilation and ICU stays than those with ARDS of other causes, whereas mortality in those patients varies considerably in different reports. Additionally, surging patients volume overwhelms hospitals bed capacity, causes staffing strains, drug and equipment supply shortages; therefore, evolving understanding of the novel pathogen and available treatment options have fueled rapid operational and clinical practice shifts in critical care^19^.

The COVID-19 pandemic crisis has been a turning point for the achievement of sustainable development goals, with all consequences at the political, economic, and socio-cultural levels. COVID-19 should be taken as an opportunity to learn from lessons taught, plan a more efficient agenda, focus on the development of additional disease-modifying interventions and adapt the actions to the current changing times in the aftermath of the pandemic^21^.

There is limited information pertaining to outcome of COVID-19 patients admitted to ICU in Greece. Accordingly, the goal of this study was to describe, for the first time, the clinical characteristics and outcomes of a cohort of mechanically ventilated patients with COVID-19 who were admitted to the intensive care in a tertiary hospital in Northern Greece. In parallel, we performed a thorough review of the literature concerning the factors that affect the ICU outcome in this category of patients worldwide.

In this paper, we present the methodology and the study population, then the results from the statistical analyses performed. The next part is the discussion section, with special analysis of the pandemic waves and viral variants of concern, the admission clinical and laboratory data, the treatment regimens and the complications during ICU stay. The limitations of the study are also discussed there, and the paper ends with the conclusion of the study and the references used.

## Materials and methods

### Patients and study design

The study was carried out through a prospective design in a single center ICU in Regional Hospital “G. Papanikolaou”, Thessaloniki, Greece. The ICU has a capacity of 18 beds and during the study operated as a COVID-19 ICU.

The study was based on the collection of data obtained during routine clinical practice in patients over 18 years old who were tested positive for SARS-CoV-2 infection by polymerase chain reaction (PCR) test between April 2020 and February 2022 (RNA extraction and RT-PCR using Extralab and Amplilab machines, respectively, by Adaltis, Italy). All the patients were intubated due to acute respiratory insufficiency and received Invasive Mechanical Ventilation.

All methods were performed in accordance with the relevant guidelines and regulations for observational studies in human subjects.

### Data collection

On day 1 at ICU admission, the following data were recorded: patient demographics and characteristics, including age, sex, body mass index (BMI), comorbidities (Diabetes mellitus, Arterial Hypertension, Chronic Ischemic Heart Disease, Chronic Kidney Disease, Chronic Obstructive Pulmonary Disease, Oncology or haematological diseases, Chemotherapy in the previous 6 months, Autoimmune diseases and Hypothyroidism), smoking status, number of days from hospital admission to intubation.

Additional data that were recorded on the same day were:

- basic laboratory values and inflammatory markers, namely white blood cells count, lymphocyte number, platelets number, urea, creatinine, Lactate Dehydrogenase (LDH), troponin, C-reactive protein (CRP), ferritin, D-Dimer and procalcitonin (PCT).
- PaO_2_/FiO_2_ ratio, SpO_2_ (from pulse oximetry), Acute Physiology and Chronic Health Evaluation (APACHE II) score, Sequential Organ Failure Assessment (SOFA) score.

During hospitalization the use of specific treatments such as azithromycin, hydroxychloroquine, dexamethasone, remdesivir and tocilizumab was recorded.

Development of important complications such as need for vasopressors, acute heart failure, renal failure, renal replacement therapy, hepatic insufficiency, acute intestinal failure, thromboembolic events, pulmonary barotrauma (pneumothorax, pneumomediastinum) was recorded during the ICU stay.

The allocation of the cases into discrete disease waves was based on the local peaks of disease outbreaks and was defined as follows: the 1st pandemic wave from March to September 2020, the 2nd from October 2020 to January 2021, the 3rd from February 2021 to June 2021 and the 4th from July 2021 to March 2022.

After the patient’s discharge from ICU or death, the length of ICU stay was calculated.

### Outcomes

The primary outcome was ICU mortality. Secondary outcomes were 28-day mortality and independent predictors of mortality at 28 days and during ICU hospitalization.

### Statistical methodology

Kolmogorov–Smirnov test was used for exploring the normality of distributions. When continuous variables had normal distribution, t-test was used for means comparison between two groups and one-way ANOVA (with Bonferroni post-hoc test) for multiple comparisons. When the variables distribution was not normal, comparisons between two groups were performed using the Mann–Whitney test. Comparisons between discrete variables were made using the x^2^ test, whereas the binary logistic regression was employed for the definition of factors affecting survival inside the ICU and 28-days survival.

The statistical analysis was performed utilizing the SPSS v21 (IBM Corp) statistical software. Differences were considered statistically significant when p ≤ 0.05.

### Ethics approval and consent to participate

The study was approved by the Scientific Council of “G. Papanikolaou” General Hospital of Thessaloniki, Greece. The observational nature of the study and the practical problems posed by the national prohibition of relatives’ visit to the ICU during the COVID-19 pandemic, led to the absence of written consent for patients’ participation to the study, which was also waived by the aforementioned ethics committee.

## Results

**Figure 1:**
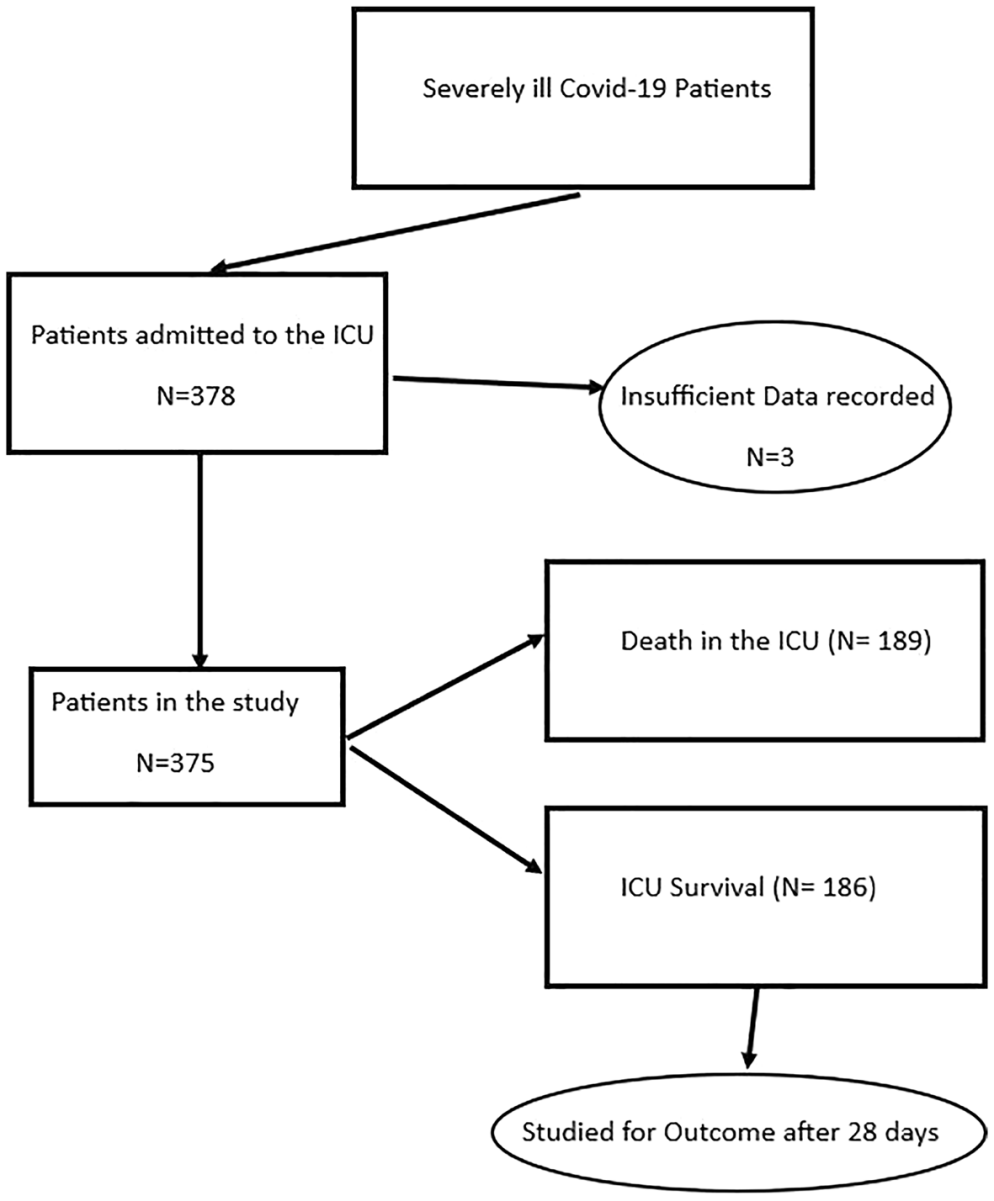
Schematic representation of the study population and outcomes.

A total of 375 patients were hospitalized in the 1^st^ ICU of “G. Papanikolaou” Hospital of Thessaloniki, Greece during the Covid-19 pandemic from the beginning of 2020 until March 2022. Of those, 239 (63.7%) were male. Figure 1 shows the general structure of the study population. The patients had a mean age of 64.1 years and had severe ARDS upon admission to the ICU with an average APACHE II of 16.3 and an average length of stay in the unit of 18 days. Table 1 shows the basic descriptive statistics of the continuous variables of the study.

**Table 1:** Descriptive statistics for the continuous variables.

Descriptive statistics					
	N	Minimum	Maximum	Mean	Std. deviation
Hospital days before ICU admission (d)	372	0	45	5.4	6
ICU LOS (d)	375	1	99	18.0	11.6
Age (y)	375	19	87	64.1	11.5
APACHE II	371	4	50	16.3	6.9
SOFA_day1	370	2	17	7.3	1.9
PaO2/FiO2_initial (mmHg)	357	33	550	100.3	64.8
SaO2_initial (%)	315	50	100	90.4	8
Ferritin (ng/mL)	300	61.00	16,500	1372.4	2021.1
IL6 (pg/mL)	10	5.0	2905	406.4	897.3
d-DIMER (μg/mL)	367	0.2	133	5.8	14.8
PCT (ng/mL)	361	0	75	1.6	6.9
CRP (mg/dL)	53	2	473	27.8	64
Urea (mg/dL)	371	14	344	61.3	43.2
Creatinine (mg/dL)	371	0.27	13.58	1.1	1.2
WBC (n/μL)	371	700	270,900	12,997.3	15,000.8
LYMPH (n/μL)	365	17	250,000	1607.9	13,085.6
PLT (n/μL)	371	311	1,070,000	267,402.2	119,928.1
TPI (pg/mL)	334	0	56,541	289.1	3332.3
LDH (u/L)	371	115	5195	639.2	446.5

*ICU LOS* ICU length of stay, *APACHE II* Acute Physiology and Chronic Health Evaluation, *SOFA* Sequential Organ Failure Assessment, *SaO2_initial* oxygen saturation of hemoglobin upon admission, *IL6* interleukin-6, *PCT* procalcitonin, *CRP* C-reactive protein, *WBC* white blood cells, *LYMPH* lymphocytes count, *PLT* platelets count, *TPI* troponin level, *LDH* lactate dehydrogenase.

Forty-four subjects were hospitalized during the first wave of the pandemic in Greece, 104 during the 2nd, 87 during the 3rd and 140 during the 4th. They were affected by the following SARS-CoV2 variants: 122 by Alpha, 165 by Beta, 78 by Delta and 10 by Omicron. In total, the ICU survival was 49.6%, whereas the 28-day survival reached 46.9%. The survival rates inside the ICU for the four main viral variants were 54.9%, 50.3%, 39.7% and 50% for the Alpha, Beta, Delta and Omicron variants, respectively. The differences in these rates were not statistically significant. Regarding the survival rates in relation to the different waves of the pandemic in Greece, the following values were observed: 61.4%, 50.96%, 62% and 37.1% for the 1st, 2nd, 3rd and 4th wave, respectively. The x^2^ test showed significant difference (p = 0.001) between the waves. Only 5 (1.3%) of the patients were fully vaccinated upon admission in the ICU in our cohort.

The sex had no significant difference in survival in the ICU or the mortality at 28 days (male 28-days survival = 46.4%, female 28-days survival = 47.8%).

**Figure 2:**
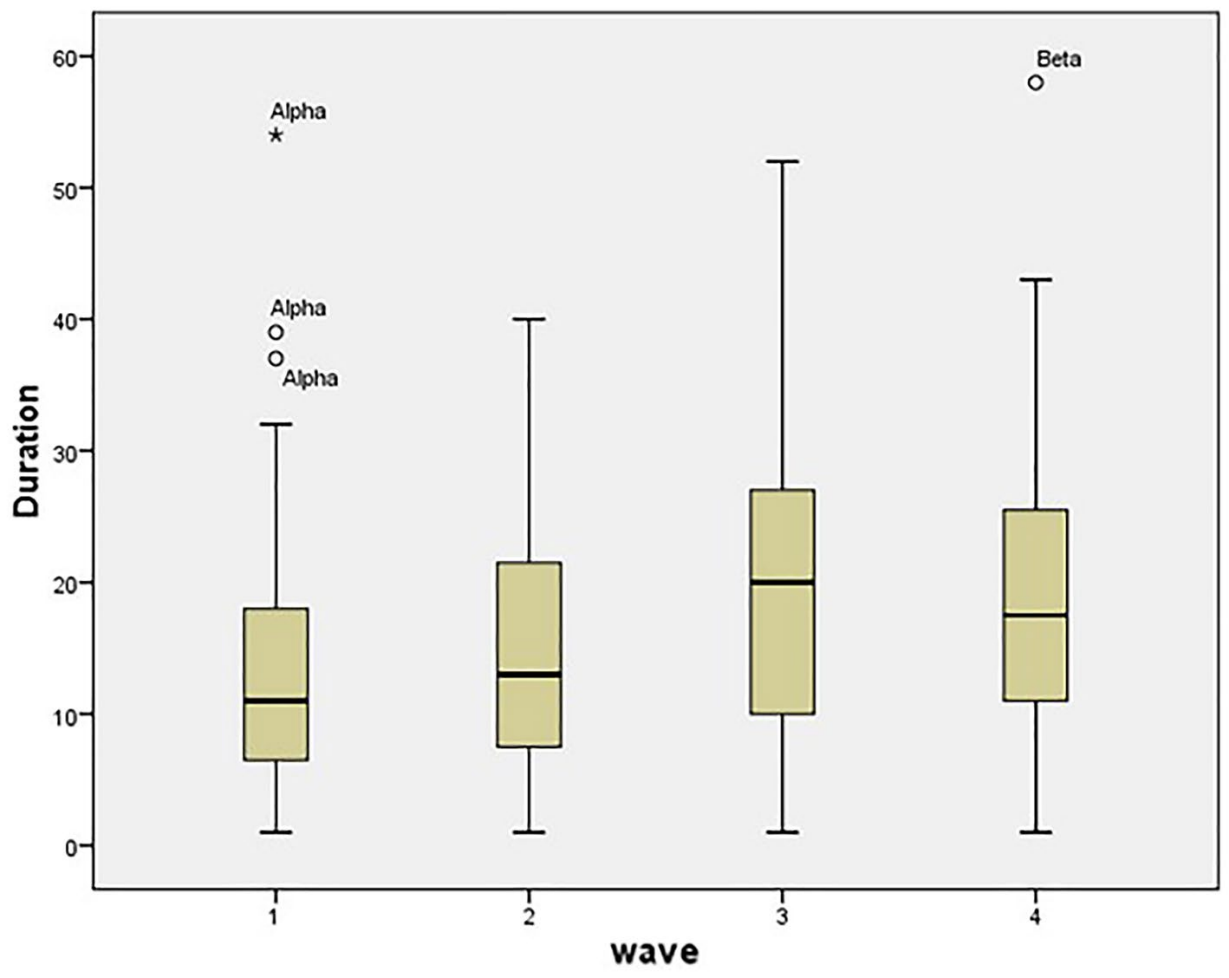
Bar chart showing the mean duration of ICU stay in relation to the Covid-19 wave in the Greek healthcare system.

Figure 2 presents the duration of ICU stay of patients in the four discrete pandemic waves.

The Kolmogorov–Smirnov test marked only the IL-6 and the PLT value as variables having normal distribution. The Mann–Whitney test for the comparison of continuous variables between patients who died in the ICU and those who survived yielded the following statistically significant results (Table 2):

**Table 2:** Mann–Whitney test for differences in various variables between non-survivors and survivors in the ICU.

Parameter	Mean value (death)	Mean value (survival)	Mean difference (death − survival)	p value
Duration of ICU stay (d)	16.7	19.4	−2.7	0.044
Age (y)	67.1	61.2	5.9	< 0.001
APACHE II	18.67	13.95	4.72	< 0.001
SOFA day 1	7.9	6.7	1.2	< 0.001
Day of ICU entry (d)	6.74	4.02	2.72	< 0.001
Ferritin (ng/mL)	1734	1029	705	< 0.001
D-dimer (μg/mL)	8.5	2.9	5.6	< 0.001
Urea (mg/dL)	73.9	48.3	25.6	< 0.001
Creatinine (mg/dL)	1.38	0.84	0.54	< 0.001
WBC (n/μL)	13,264	12,722	542	0.001
TPI (pg/mL)	520.5	40.3	480.2	< 0.001
LDH (u/L)	712	563	149	0.001

*APACHE II* Acute Physiology and Chronic Health Evaluation, *SOFA* Sequential Organ Failure Assessment, *WBC* white blood cells, *TPI* troponine level, *LDH* lactate dehydrogenase.

Moreover, patients who were treated with remdesivir had shorter ICU stay (4.74 vs 5.84 days, p = 0.014) and had better survival rates during the ICU stay (62.5% vs 41.15%, p < 0.001) as well as at 28 days.

The ANOVA tool with Bonferroni post-hoc tests among the four virus variants showed significant differences in:

- Length of ICU stay: Beta–Alpha = 4.7 days & Delta–Alpha = 13.2 days
- Age: Beta–Delta = 4.54 years
- d-dimer: Delta–Alpha = 13.7 & Delta–Beta = 13.2

Concerning the x^2^ tests for comparisons between discrete variables, the following noteworthy results were obtained:

- Patients with acute kidney injury (AKI), coronary disease, enteral insufficiency, heart failure, liver insufficiency and barotrauma had worse outcome in the ICU.
- The same conditions had similar effect to the 28-days survival.
- Smokers had better outcomes in terms of ICU and 28-days survival.
- The wave of the pandemic played an important role in survival (both the ICU and 28-days) with the 1st wave having a survival rate of 61.4%, the 2nd 51%, the 3rd 62% and the 4th 37.1%.
- Tocilizumab and T-lymph therapies did not show significant impact on outcome.

Tables 3 and 4 show the statistically significant differences in discrete variables between survivors and non-survivors in the ICU and at 28 days, based on the x^2^ tests.

**Table 3:** Differences in discrete variables in relation to ICU survival (x^2^ tests).

Parameter	Difference in survival	p value
Remdesivir	62.5% vs 41.15%	< 0.001
Smoking	73.3% vs 46.4%	0.001
Coronary disease	38.5% vs 52.3%	0.029
CKD	27.8% vs 52.1%	0.004
Acute kidney injury	21.4% vs 71.3%	< 0.001
Liver insufficiency	25% vs 52.5%	0.002
Cardiac failure	23.4% vs 56%	< 0.001
Enteral insufficiency	24% vs 68.5%	< 0.001
Barotrauma	34% vs 52%	0.013
Sepsis	53.6% vs 38%	0.01

*CKD* chronic kidney disease.

**Table 4:** Differences in discrete variables in relation to 28 days-survival (x^2^ tests).

Parameter	Difference in survival	p value
Remdesivir	60.4% vs 38.5%	< 0.001
Smoking	68.9% vs 44.3%	0.002
Coronary disease	38.5% vs 52.3%	0.022
CKD	25% vs 49.7%	0.004
Acute kidney injury	19.5% vs 68%	< 0.001
Liver insufficiency	25% vs 49.8%	0.005
Cardiac failure	20.3% vs 53.7%	< 0.001
Enteral insufficiency	22.7% vs 65.3%	< 0.001
Barotrauma	28% vs 49.8%	0.003
Antifungal therapy	40.4% vs 58.4%	0.022

*CKD* chronic kidney disease.

It is of interest to note that 50 patients (13.3%) developed barotrauma during the ICU stay. One additional parameter approached statistical significance in 28-days survival: hypothyroidism (31% vs 48.4%, p = 0.053).

Logistic regressions for outcome revealed that the following parameters were independently associated with ICU survival: wave, SOFA day1, remdesivir use, AKI, enteral insufficiency, sepsis, duration of ICU stay and WBC.

Similarly, by applying binary logistic regression, we found that the parameters affecting the 28-days survival were: duration of stay in ICU, SOFA day1, WBC, Wave, AKI and Enteral Insufficiency. Two additional parameters were close to achieving statistical significance: remdesivir and development of barotrauma.

Concerning the statistical power of the sample size, it was discovered that for a difference of average mortality of approximately 6% (which was the case in our population compared to the literature) and an equal distribution between death and survival, a sample size of 380 subjects would yield a statistical power of 80%.

## Discussion

This study identified the association of the characteristics of pandemic (waves, virus strains responsible for infection), baseline patient characteristics, clinical and laboratory data on admission and during ICU stay with 28-day mortality and mortality at ICU discharge in patients with SARS COV-2 admitted to the ICU of a tertiary hospital. This is the first study designed to describe clinical characteristics and outcomes of COVID-19 patients with ARDS receiving prolonged mechanical ventilation from a large Northern Greece area.

Recognizing the variables that independently predict death in COVID-19 is of great importance. Logistic regressions for outcome revealed that the following parameters were independently associated with 28-day and ICU survival: wave sequence, SOFA score on admission, the use of Remdesivir, presence of AKI, presence of gastrointestinal failure, and WBC values/levels. In addition, sepsis influenced ICU survival but not 28-day survival, a finding in agreement with recent literature^22^. The use of remdesivir and development of barotrauma were close to achieving statistical significance in affecting 28-day outcome, however no association was observed in relation with the ICU mortality rate.

### Waves, viral variants of concern (VOCs) and outcome

The 28-day survival rate of 46.9% was demonstrated, whereas the overall survival rate upon ICU discharge reached 49.6%. Statistically significant differences in ICU mortality were observed among patients admitted during the different waves of the pandemic: the mortality rate was significantly higher in patients admitted to the ICU during the first and third waves of the pandemic.

Diverse COVID-19 associated mortality rates have been reported from different studies among critically ill patient populations during the beginning of the pandemic. During the first wave of the pandemic, the mortality rates of patients with COVID-19 treated in ICUs ranged broadly (23.3–81%)^23–28^.

A multicentre retrospective cohort study of Carbonella et al. referred to overall ICU mortality of 30.7%, without significant differences between study periods (first wave 31.7% vs second/third waves 28.8%, p = 0.06). After adjusting for confounding factors through a multivariable analysis, no significant association was found between the COVID-19 waves and mortality (OR 0.81, 95% CI 0.64–1.03; p = 0.09)^29^.

The impact of waves sequence on patients’ outcomes during the course of the pandemic around the world could be difficult to analyze and interpret due to the fact that in different regions pandemic waves occurred in different periods of time, patients included in studies had diverse underlying medical conditions and diverse severity of illness and need for invasive mechanical ventilation^30–32^.

Recently published studies have demonstrated the association between increased mortality and hospital load on critical care capacity^30,33^. Possible explanations for the higher mortality during the first waves in comparison with the fourth wave may have to do with the insufficient knowledge of the disease during the early phases of pandemic, as well as the claimed inability of the health systems to control the unprecedented ICU capacity strain resulting from sharp increase in patient volume during the peaks of the initial waves^29^. ICU capacity strain can be characterized as a mismatch between supply of recourses including beds, staff, and/or other resources and demand (the need to admit and provide care for critically ill patients^34^). ICU capacity strain has adverse consequences for patient outcomes but, additionally, may has an adverse effect on the well-being of the healthcare providers by increasing stress levels^35,36^. It is imperative to investigate how excessive ICU demand may negatively affect patients’ outcome and to identify regulatory strategies to prevent healthcare system overload associated with high mortality.

The lack of some effective antiviral treatment could be considered as an additional factor affecting the mortality rate in our patients, as our results revealed significant increase on the use of antivirus therapy (remdesivir) during the fourth wave in comparison with the previous periods of pandemic.

Our data demonstrated that the ICU survival rate did not differ significantly when infection with the four main VOCs was considered. However, The Bonferroni post-hoc tests among the four virus variants showed significant differences in the length of ICU stay among different VOCs: patients with Beta and Delta variants had longer duration of stay in comparison with patients with Alpha variants.

Owing to a possible risk of increasing the transmissibility of the virus, severity of the infected individuals, and the ability to escape the antibody produced by the vaccines, the four SARS-CoV-2 variants of Alpha (B.1.1.7), Beta (B.1.351), Gamma (P.1), and Delta (B.1.617.2) have attracted the most widespread attention. However, the conclusions regarding disease severity and impact on outcome of these variant viruses are not consistent in the literature. A recent review showed that variants Alpha, Beta and Gamma all had a higher risk of hospitalization and ICU admission compared with the wild-type virus, with the higher risk for Beta variant^37^. A systematic review and meta- analysis study of Lin et al. aimed to determine the effects of SARS-CoV-2 variants of concern on disease severity and clinical outcomes. The analysis showed that all SARS-COV-2 VOCs have a higher risk of disease severity than the wild-type virus. By comparing with the wild-type virus, in terms of the risk of hospitalization, ICU admission, and mortality, the variants Beta and Delta have a higher risk than the variants Alpha and Gamma, however, Alpha variant had a higher risk of disease severity than the wild-type virus^38^. The results showed that in the risk of hospitalization, ICU admission, and mortality, all the SARS-CoV-2 VOCs had different degrees of increase compared with wild-type virus; Delta variant had the highest risk of ICU admission and mortality, and Beta variant had the highest risk of hospitalization. Contrastingly, Funk et al. found that Alpha variant was more threatening than the wild-type virus and associated with significantly higher risk of hospitalization and ICU admission^39^.

Moreover, consistently with our results, a meta-analysis study of Kow et al. demonstrated significantly increased hazard of mortality among patients with COVID-19 infected with Alpha variant relative to those infected with the wild-type virus^40^. Although, the phenotypic effects of SARS-CoV-2 VOCs are still uncertain and the emergence of advanced vaccines may reduce the threat posed by SARS-CoV-2 variants, the impact of VOCs on outcome of patients seems to be important. Of course, other than VOCs factors, including the use of health-care resources, demographic changes, strain posed on healthcare system possibly influenced clinical outcomes in our patients and lead to a higher mortality rate in patients with the Alpha variant.

### Admission clinical and laboratory data and outcome

Our results showed that non survivors were older, had shorter duration of ICU stay, higher APACHE II score and SOFA scores on admission, higher levels of laboratory markers, including ferritin, d-dimer, urea and creatinine levels, white blood cells (WBC), troponin (TPI) and lactate dehydrogenase (LDH).

Nicholson et al. evaluated the risk factors on admission (including comorbidities, vital signs, and initial laboratory assessment) associated with need for mechanical ventilation and in-hospital mortality in COVID-19 patients. Older age, male sex, coronary artery disease, diabetes mellitus, chronic statin use, SpO_2_/FiO_2_ ratio and body mass index, high levels of lactate dehydrogenase and high levels of inflammatory and infectious biomarkers (neutrophil to lymphocyte ratio, platelet count, procalcitonin, C-reactive protein) were identified as important risk factors for mechanical ventilation requirement and in-hospital mortality. Using these factors, the authors constructed specific risk scores for healthcare providers and researchers^41^.

The higher severity of illness and severity of organ failure and the excessive levels of inflammatory and infectious markers in our patients confirms the association of aggressive inflammatory response leading to organ dysfunction and the higher mortality rate^42^. Observational research published at the beginning of the pandemic suggests that the risk of mortality increases with the presence of comorbidities: obesity, hypertension, diabetes mellitus, and chronic lung disease^43^.

It has been inferred that patients with cardiac injury (elevated TPI) have a worse prognosis, suggesting specific target organ damage by SARS-CoV-2^44^. Preexisting renal and cardiovascular disease may increase the risk of a hyperinflammatory response that amplifies the effect of viral infection^45,46^.

Underlying kidney disease is an emerging risk factor for more severe coronavirus disease 2019 (COVID-19) illness. Data from our study confirm previous reports about high mortality of patients with underlying kidney disease and severe COVID-19, highlighting the importance of identifying safe and effective treatment modalities in this vulnerable patients’ population^47,48^. In our study patients with coronary artery disease and high levels of troponin demonstrated higher mortality rate, however the presence of other evaluated co-morbidities (diabetes mellitus, arterial hypertension, COPD) did not have a significant impact on outcome.

A study of Guan et al. performed in COVID-19 patients found that arterial hypertension was the most prevalent comorbidity (16.9%), followed by diabetes (8.2%), cardiovascular disease (3.7%), cerebrovascular disease (1.9%), COPD (1.5%) and malignancy (1.1%). In the Cox regression model, after adjustment for age and smoking status, the independent risk factors associated with the composite outcome were malignancy, COPD, diabetes and hypertension^49^. A meta-analysis study of Roncon et al. demonstrated increased risk of ICU admission (OR: 2.79, 95% CI 1.85–4.22, p < 0.0001, I^2^ = 46%) and higher mortality risk in patients with diabetes (OR 3.21, 95% CI 1.82–5.64, p < 0.0001, I^2^ = 16%)^50^. Espiritu et al. in a nationwide cohort study, involving 37 hospitals showed longer duration of ventilator dependence, longer length of hospital stay and increased risk of mortality in diabetic patients with COVID-19 infection^51^. Our results did not confirm these data and contrasted with the results of other studies which showed an association of diabetes mellitus with poor outcome in patients with COVID-19.

A recent umbrella systematic review and meta-analysis study that evaluated the impact of diabetes on mortality and hospital outcomes in COVID-19 patients highlighted significant data variability across world regions and major worldwide discrepancies and data variability in major clinical outcomes which may skew overall trends in these complicated relationships. Whether this finding comes as a result of the variability of healthcare provisions for management of patients with diabetes remains to be fully elucidated^52^.

Interestingly, our data demonstrated that despite the fact that COPD diagnosis did not affect the survival rate, smokers had better 28-day and ICU outcome. A study of Meza et al. analyzed information from 81 academic hubs shared within National COVID Cohort Collaboration (N3C) database on COVID-19 clinical data and revealed higher odds ratio of mortality in patients who had COPD compared with those who did not, even after adjusting for other known risk factors [2.1 (1.96, 2.26, p-value < 0.001)]^53^. However, in accordance with our findings, a study from Italy, performed in four centers showed that the prevalence of COPD and current smokers in patients with was not high, suggesting that they were not at increased risk of getting the infection. Although when SARS-CoV-2 infection occurred, COPD patients and former smokers with SARS-CoV-2 infection were those with the highest all-cause mortality, this high mortality rate seemed to be mainly related to the presence of comorbidities and not to COPD and smoking itself^54^.

Our results revealed a marginal association of hypothyroidism with patient outcome at 28 days. Studies investigating the relationship between thyroid disease and COVID-19 outcomes demonstrated that the presence of unspecified thyroid disease and hypothyroidism on admission were associated with poor outcomes^55^. It is suggested that COVID-19 infection triggers the activation of pre-existing thyroid disease; the relationship of COVID -19 and thyroid function have been linked to both direct effect of infection (caused by viral infection of target cells) and indirect effect (caused by abnormal immune-inflammatory responses to the virus, involving “cytokine storm”-mediated autoimmune effect)^56^. However, a large retrospective Study of Van Gerwen et al. did not confirm the association between outcome and the presence of hypothyroidism and suggested no additional precautions for these patients^57^. Future research into the potential complications of COVID-19 on the thyroid gland and function is warranted.

Interestingly, we observed extremely low rate of vaccination in our patients’ cohort, only 5 of the patients were fully vaccinated upon admission in the ICU. The small number of vaccinated patients has not allowed us to draw clear conclusions about the association of vaccination with outcome in our patients’ population.

### Treatment regimens and outcome

Patients who were treated with remdesivir had shorter ICU stay (4.74 vs. 5.84 days, p = 0.014) and had better ICU and 28-day survival rates (62.5% vs. 41.15%, p < 0.001 and 60.4% vs 38.5%, p < 0.001, respectively). Data from the literature show that remdesivir was superior to placebo in shortening the time to recovery in adults who were hospitalized with Covid-19 and had evidence of lower respiratory tract infection. Results of one of the largest study focused on the effectiveness of remdesivir (ACTT-1 study, double-blind, randomized, placebo-controlled trial) showed that remdesivir was superior to placebo in shortening the time to recovery in adults who were hospitalized with Covid-19 and had evidence of lower respiratory tract infection^58^. This study demonstrated that patients who received remdesivir had a median recovery time of 10 days, as compared with 15 days among those who received placebo (rate ratio for recovery, 1.29; 95% CI, 1.12 to 1.49; _P_ < 0.001). Additionally, this study showed that the patients who received remdesivir were found to be more likely to have clinical improvement at day 15 than those who received placebo (odds ratio, 1.5; 95% CI, 1.2 to 1.9, after adjustment for actual disease severity). The Kaplan–Meier estimate of mortality revealed lower mortality by days 15 and 29 in patients who received remdesivir. However, data from the recently published randomized trial enrolling 14,221 patients from 454 hospitals in 35 countries (Solidarity study) combined with updated meta-analysis (of mortality in randomised trials of remdesivir versus no remdesivir) revealed no significant effect on patients with COVID-19 who were already under mechanical ventilation. Among other hospitalized patients, it has a small positive effect on mortality or progression to ventilation (or both). Additional meta-analyses of all randomized trials showed that their results are closely consistent with Solidarity’s outcome findings^59^. Various immune modulators, and monoclonal antibodies against currently circulating VOCs are now emerging that might prove more effective than remdesivir infusions, but large-scale randomised evidence will be warranted to evaluate and compare them.

Tocilizumab, a monoclonal antibody against the interleukin 6 receptor, may counteract the inflammatory cytokine release syndrome in patients with severe COVID-19 illness. Data from literature demonstrated that among critically ill patients with COVID-19, early treatment with tocilizumab may reduce mortality and requirement for mechanical ventilation^60–63^. In a randomised, double-blind, placebo-controlled trial of tocilizumab in hospitalized patients with severe Covid-19 pneumonia (COVACTA study), the use of tocilizumab did not result in significantly better clinical status or lower mortality than placebo at 28 days. Although there was no difference in mortality between tocilizumab and placebo, potential benefits in time to discharge and duration of intensive care unit (ICU) stay were identified^64^. Our study findings confirm these data and did not demonstrate any advantage from the use of Tocilizumab regarding outcome parameters.

Cell therapies offer great potential in treating severe COVID-19 presentations due to their customizability and regenerative function. Cell therapies have the potential to regenerate damaged tissue and trigger the immune system and, hence, are a treatment option with great promise. However, the inclusion of T-lymph therapies in the treatment regimen was not associated with significant impact on outcome. Future investigations may explore potential biomarkers to optimize patient selection for tocilizumab treatment and combination therapy with various cell therapies.

Severely ill coronavirus disease-19 (COVID-19) patients, admitted to intensive care units (ICUs) are at increased risk of fungal secondary infections. Invasive candidiasis, pulmonary aspergillosis, and mucormycosis are the most frequently reported fungal secondary infections, associated with increased morbidity and mortality in COVID-19 patients^65,66^. The commonly used antifungal therapy (AFT) includes liposomal amphotericin B, azoles, and echinocandins^67^. A systematic review and meta-analysis investigating the use of antifungal therapy in the management of fungal secondary infections in COVID-19 patients revealed high prevalence of fungal secondary infections among COVID-19 patients (28.2%). Meta-analysis results of this study found that all-cause mortality in COVID-19 patients with fungal secondary infections is not significantly associated with type and duration of AFT, mostly due to presence of confounding factors such as small number of events, delay in diagnosis of fungal secondary infections, presence of other co-infections and multiple comorbidities^68^. An observational study by Hatzl et al. demonstrated that in ICU patients with COVID-19, antifungal prophylaxis was associated with significantly reduced COVID-19-Associated Pulmonary Aspergillosis (CAPA) incidence, but this did not translate into improved survival. The use of antifungal treatment was associated with higher mortality rate at 28-day (p = 0.022), however, the association with ICU outcome was not statistically significant^69^. Randomized controlled trials are warranted to evaluate the efficacy and safety of antifungal prophylaxis with respect to Coronavirus disease 19 -associated fungal infections incidence and clinical outcomes.

### Complications during ICU stay and outcome

Organ damage in COVID-19 patients has been of special concern. Our results demonstrated the association of acute kidney injury (AKI), acute hepatic and acute gastrointestinal injuries with outcome among COVID-19 patients. Organ dysfunction observed in our patients could be caused by a generalized inflammatory response to SARS-CoV-2, affecting multiple organ systems.

Statistical analysis revealed significant differences in the following complications in relation to both 28-day and ICU survival: the presence of acute kidney injury, acute cardiac failure, acute hepatic failure and acute gastrointestinal injury, sepsis and presence of barotraumas during ICU stay. Acute kidney injury is common among critically ill patients with COVID-19 and is associated with worse prognosis and a high risk of developing chronic kidney disease (CKD)^70,71^. A large UK cohort study demonstrated a high AKI incidence; 487 (39%) out of 1248 patients experienced AKI. Acute kidney injury was a strong predictor of 30-day mortality with an increasing risk across AKI stages^72^. Data from a multicenter retrospective cohort analysis of patients with critical COVID-19 in seven large hospitals in Belgium confirm this association and demonstrated even higher incidence of AKI in critically ill patients approaching 80%; additionally, after multivariable adjustment, all AKI stages were associated with ICU mortality^73^. In our study the presence of acute kidney injury was associated with higher ICU and 28-day mortality rate; the rate of AKI in non survivors was 71.3% compared to 21.4% in survivors. Moreover, AKI diagnosis was independently associated with ICU survival.

Logistic regressions for outcome revealed that the following parameters were independently associated with ICU survival: wave, SOFA day1, Remdesivir use, AKI, enteral insufficiency, ICU length of stay and WBC.

The association of acute gastrointestinal injury and mortality among COVID-19 patients was not sufficiently elucidated. The development of high-grade acute gastrointestinal injury (AGI) and feeding intolerance (FI) during ICU stay can serve as a prognostic tool to predict outcomes in critically ill COVID-19 patients, however there is a relative scarcity of data regarding the impact of AGI on outcome COVID-19 patients. Initial data from Wuhan, China reported a high incidence of AGI in patients with COVID-19 disease; diagnosis of AGI was associated with a higher incidence of septic shock and 28-d mortality^74^. Drakos et al. found extremely high incidence of AGI and feeding intolerance among critically ill COVID-19 patients. The overall incidence of AGI was 95% (45% AGI I/II, 50% AGI III/IV), and FI incidence was 63%. Patients with AGI III/IV were more likely to have prolonged mechanical ventilation (22 days vs 16 days, p-value < 0.002) and higher mortality rate (58% vs 28%, p-value < 0.001) compared to patients with AGI 0/I/II^75^. Our findings are consistent with conclusions from the previous publications. We categorized AGI into four grades based on collected clinical and imaging data. Logistic regression analysis performed in our study demonstrated that the presence of AGI grades III and IV was associated with higher 28-day and ICU mortality rates. Consequently, a conclusion could be made that presence of AGI III/IV may reflect the degree of inflammatory response and multiple organ damage as reflected by higher WBC levels and higher SOFA score in this patient population, which has been shown to correlate with disease mortality.

A high rate of barotraumas was observed in our patients’ population: 50 patients (13.3%) developed barotrauma during the ICU stay; however, the logistic regression analysis failed to show a strong relationship between presence of barotraumas and outcome. An ever-increasing number of studies have reported an increased incidence of spontaneous pulmonary barotrauma such as pneumothorax, pneumomediastinum, and subcutaneous emphysema in patients with COVID-19^76,77^. A systematic review and meta-analysis aimed to evaluate the incidence of barotraumas and impact on outcome showed a high incidence 18.4% (13–25.3%) in patients receiving invasive mechanical ventilation. In addition, barotrauma was associated with a longer length of ICU and hospital stay, and higher in-hospital mortality. When compared with non-COVID-19 ARDS, a slightly higher odds of barotrauma were seen in COVID-19 ARDS compared with non-COVID19 cases^77^. Further studies are required to unravel the underlying pathophysiology and develop safer management strategies.

### Limitations

This study has several limitations. First, the study was conducted in the Northern Greece area, therefore, results from our study may not be representative of other regions in Greece and may not be extrapolated to other world regions. Second, the observational nature of the study implies that a number of possible unmeasured confounders could affect the outcomes. Further study into possible differences in the provision of care and outcome for COVID-19 and non-COVID-19 patients is needed. Third, longer follow up such as hospital mortality were not evaluated. Reporting short-term endpoints could be not enough and the investigation of long-term outcomes can be highly important in critical care settings. Fourth, we focused on all-cause ICU mortality. To be able to better interpret mortality rates, more data of the specific causes of death in COVID-19 would be helpful.

## Conclusion

Recognizing the variables that independently predict death in COVID-19 is of great importance. In this observational cohort study of 375 COVID-19 patients we report an association between clinical and laboratory data with outcome. Strength of this study is the large number of patients with severe form of coronavirus disease who were all under invasive mechanical ventilation. Overall, 90-day mortality was 49.6%, the wave of the pandemic played an important role in survival which was constantly decreasing over time during the study period. Comparison of the adjusted mortality rates between pandemic waves within a two year-study period adds additional understanding and additional potential and tools for pandemics’ management. Recognizing the variables that independently predict death in COVID-19 is of great importance for pandemic management. Logistic regressions for outcome revealed that the following parameters were independently associated with ICU survival: wave, SOFA on day1, remdesivir use, acute kidney injury, sepsis, presence of gastrointestinal failure, duration of ICU stay and WBC. Similarly, the parameters affecting the 28-days survival were: duration of stay in ICU, SOFA on day1, WBC, wave, acute kidney injury and presence of gastrointestinal failure. Our results did not confirm data from the previous publications regarding the association of comorbidities (diabetes mellitus, arterial hypertension, COPD) with poor outcome in patients with COVID-19. Our results revealed a marginal association of hypothyroidism with patient outcome at 28 days. Future research into the potential complications of COVID-19 on the thyroid gland and function is warranted. One of the strengths of the study include the analysis of relationship between immunotherapy treatment and patients’ outcome. Despite many references that advancing novel therapeutic developments becomes crucial to minimize the number of deaths from COVID-19, the use of Tocilizumab and T-lymphocyte therapy did not demonstrate any advantage regarding outcome parameters in our study population. Future investigations may explore potential biomarkers to optimize patient selection for tocilizumab treatment and combination therapy with various cell therapies.

## Data Availability

The datasets used and/or analyzed during the current study are available from the corresponding author on reasonable request.

## Abbreviations

APACHE II: Acute Physiology and Chronic Health Evaluation Score
SOFA: Sequential Organ Failure Assessment
WBC: White blood cells
TPI: Troponin level
LDH: Lactate dehydrogenase
VOC: Variants of concern
ICU: Intensive care unit
COVID-19: Corona virus disease 2019
ARDS: Acute respiratory distress syndrome
CKD: Chronic kidney disease
AKI: Acute kidney injury
AGI: Acute gastrointestinal injury
FI: Feeding intolerance
CAPA: COVID-19-associated pulmonary aspergillosis
AFT: Anti fungal therapy
IL-6: Interleukin-6
PLT: Platelets count
COPD: Chronic obstructive pulmonary disease
PCR: Polymerase chain reaction
BMI: Body mass index
CRP: C-reactive protein
